# Neural Evidence of Language Membership Control in Bilingual Word Recognition: An fMRI Study of Cognate Processing in Chinese–Japanese Bilinguals

**DOI:** 10.3389/fpsyg.2021.643211

**Published:** 2021-06-07

**Authors:** Ming-Che Hsieh, Hyeonjeong Jeong, Motoaki Sugiura, Ryuta Kawashima

**Affiliations:** ^1^Graduate School of International Cultural Studies, Tohoku University, Sendai, Japan; ^2^Institute of Development, Aging and Cancer, Tohoku University, Sendai, Japan; ^3^International Research Institute of Disaster Science, Tohoku University, Sendai, Japan

**Keywords:** cognates, interlingual homographs, stimulus list composition, bilingualism, fMRI

## Abstract

This study aims to examine the neural mechanisms of resolving response competition during bilingual word recognition in the context of language intermixing. During fMRI scanning, Chinese–Japanese unbalanced bilinguals were required to perform a second-language (L2) lexical decision task composed of cognates, interlingual homographs, matched control words from both Chinese (first language) and Japanese (L2), and pseudowords. Cognate word processing showed longer reaction times and greater activation in the supplementary motor area (SMA) than L2 control word processing. In light of the orthographic and semantic overlap of cognates, these results reflect the cognitive processing involved in resolving response conflicts enhanced by the language membership of non-target language during bilingual word recognition. A significant effect of L2 proficiency was also observed only in the SMA, which is associated with the task decision system. This finding supports the bottom-up process in the BIA+ model and the Multilink model. The task/decision system receives the information from the word identification system, making appropriate responses during bilingual word recognition.

## Introduction

Previous bilingual studies have demonstrated that lexical information (orthography, phonology, and meaning) is activated simultaneously in both languages and competes with each other during visual word recognition (Dijkstra and van Heuven, 1998; van Heuven and Dijkstra, 2010; Hsieh et al., 2017). However, it is still controversial to determine whether language memberships enhance response competition during bilingual word recognition. Cognates are well-suited for examining such controversy because they are cross-linguistic word types that share lexical information between two languages but have two language memberships. For instance, the orthographic form “man” shares the same meaning in English and Dutch. In comparison with matched control words in either English (e.g., duck) or Dutch (e.g., end “duck”), bilinguals tend to recognize cognates faster because the shared orthographic forms and meanings facilitate bilingual word recognition. The cognate facilitation effect is among the pieces of evidence suggesting that bilinguals access both languages simultaneously (see van Heuven and Dijkstra, 2010 for a review). The cognate facilitation effect has been reported from earlier psycholinguistic studies using isolated words (e.g., Van Hell and Dijkstra, 2002; Dijkstra et al., 2010; Comesaña et al., 2012) and sentences (e.g., Duyck et al., 2007; Van Assche et al., 2012).

Bilingual Interactive Activation Plus (BIA+) model (Dijkstra and van Heuven, 2002) attempts to explain the cognate facilitation effect on bilingual word recognition by assuming that bilinguals integrate lexical information from both languages. In this model, the word identification system processes lexical information and language memberships (i.e., language nodes) in a bottom-up manner, whereas the task/decision system regulates the response, depending on task requirements. Because the task/decision system receives the information from the word identification system to make an appropriate response for tasks, the bottom-up process is essential in the BIA+ model. According to the BIA+ model, owing to the orthographic and semantic overlap between languages, identical cognates (e.g., “man” in English and Dutch) reduce the level of cross-linguistic competition in the word identification system. However, due to the partially orthographic overlap, non-identical cognates [e.g., olive (English) vs. olijf (Dutch)] elicit lateral inhibition between the two orthographic representations, resulting in the smaller facilitation effect than identical cognates. The task/decision system uses the information from the word identification system to select an appropriate response to an input word for a particular task or a context.

Multilink (Dijkstra et al., 2019), a subsequent localist-connectionist model of the BIA+ model, provides a general account of retrieval of orthographic form and word meaning during language comprehension and production. Identical to the BIA+ model, Multilink includes the lexical network and the task/decision system. Once lexical-orthographic representations are activated by written input, the semantic and phonological representations and language memberships are activated simultaneously through a bidirectional flow. However, different from the BIA+ model, Multilink assumes that bidirectional excitatory connections between representations involve bilingual language processing rather than lateral inhibition (i.e., lexical competition). While the lexical competition does not arise within the word identification system, the competition arises because of the response selection in the task/decision system (cf. Declerck et al., 2019). According to Multilink, the orthographic overlap of cognates activates the shared semantics between languages through a bidirectional flow between orthographic and semantic nodes. This flow causes cognates to be activated more quickly than the semantic node receiving activations from the single orthographic node (e.g., matched language control words and non-identical cognates). Therefore, without lateral inhibition, the cognate facilitation effect is led by a bottom-up process. The task/decision system, independent of the lexical network, receives the information from the lexical network and makes appropriate responses for tasks (see Dijkstra et al., 2019 for details of simulation).

Despite these previous theoretical backgrounds, recent psycho- and neurolinguistic studies have demonstrated that the cognate facilitation effect does not always emerge but rather may be modulated by contextual information (i.e., stimulus list composition) and the language proficiency of bilinguals (e.g., Brenders et al., 2011; Peeters et al., 2013; Hsieh et al., 2017). The cognitive mechanism underlying cognate processing appears to be sensitive to whether the stimuli list comprises the bilinguals’ two languages and has not yet been fully proven by behavioral and neuroimaging studies. According to context-sensitive lexical access (see Dijkstra and van Heuven, 2018 for a review), lexicons in two languages are simultaneously accessed, and contextual information allows bilinguals to select an appropriate response to a given context during word recognition (e.g., stimulus list composition). The cognate facilitation effect was mainly observed from *single language stimulus list* composed of cognates and matched control words from bilinguals’ first (L1) or second language (L2) only (e.g., Peeters et al., 2013; Xiong et al., 2020). Because no word belongs to any language other than the target language, any lexical activation may be regarded as evidence supporting facilitation in word recognition (Vanlangendonck et al., 2020). In contrast, according to context-sensitive lexical access, the composition of the stimulus list could influence participants’ language processing (Dijkstra and van Heuven, 2018). The awareness of language memberships may be enhanced by *mixed-language stimulus list composition*, including words from bilinguals’ two languages (e.g., mixing cognates, bilinguals’ L1 and L2). Therefore, bilinguals may face response conflicts when recognizing cognates in a mixed-language context, as cognates belong to two language memberships.

In order to discuss whether stimulus list composition influences bilinguals’ recognition of cognates, Poort and Rodd (2017) recruited Dutch–English bilinguals to complete two versions of lexical decision tasks: One is the single language stimulus list, which included only cognates, control words in English, and regular non-words; and the other is the mixed-language stimulus list further including interlingual homographs, control words in Dutch, and pseudohomophones (instead of regular non-words). The Dutch–English bilinguals were required to determine whether the stimuli belong to English or not by replying “yes” or “no” explicitly through button pressing. A significant cognate facilitation effect was observed from the single language stimulus list, but the disadvantage for cognates was observed from the mixed-language list. Similarly, Vanlangendonck et al. (2020) recruited Dutch–English bilinguals to complete English lexical decision tasks under the same experimental procedure as Poort and Rodd (2017). Still, the word types were slightly different between these two studies. The single language stimulus list consisted of interlingual homographs, identical cognates, non-identical cognates, English control words, and pseudowords. The mixed-language stimulus list further included Dutch control words. Their results were consistent with those of Poort and Rodd (2017), suggesting that stimulus list composition determines facilitation and inhibition effects during cognate recognition. Besides, Vanlangendonck et al. (2020) show the unique role of identical cognates since the reaction time of non-identical cognates did not differ relative to control words in both stimulus lists.

Peeters et al. (2019) demonstrated that bilinguals relied on language control areas when facing cognates in a lexical decision task with a mixed-language stimulus list composition. The stimuli and procedure were identical to those of Vanlangendonck et al. (2020). In the single language stimulus list, the activation of language control areas such as the left inferior frontal gyrus (IFG) and supplementary motor area (SMA) was observed from interlingual homographs, not from identical and non-identical cognates. However, identical cognates enhanced greater activation in the bilateral IFG and SMA in the mixed-language list. This means that the interference effect occurred when participants needed to distinguish two languages in a mixed language context. The results suggest that a link between non-target language membership of cognates and a “no” response could cancel out the facilitation effect and even further induce cognates’ response conflicts.

Manipulating different tasks, van Heuven et al. (2008) observed that response conflicts were caused by the link between representations and responses. Dutch (L1)-English (L2) bilinguals were invited to perform a general lexical decision task (GLD) and an English (L2) lexical decision task (ELD) through *single language stimulus list* composed of interlingual homographs, matched English control words, and pseudowords. In the GLD task, the participants were required to press a button when the stimuli were real words regardless of whether that word was Dutch or English. Thus, response conflicts would not occur in recognizing interlingual homographs in the GLD task. In the ELD task, where participants had to focus on English words, the lexical representations of non-target language (i.e., Dutch) were more likely to evoke a “no” response rather than a “yes” response in recognizing interlingual homographs. Therefore, response conflicts would increase in the ELD task. When the bilinguals processed interlingual homographs, the activation of the left IFG with stimulus-based conflict was observed regardless of the two tasks. However, the SMA and dorsal anterior cingulate cortex (dACC) were activated to resolve response conflicts in the ELD task only.

Similar research outcomes were observed from logographic writing systems such as Chinese characters and Japanese kanji (e.g., Hsieh et al., 2017). In Hsieh et al.’s (2017) study, Chinese (L1)-Japanese (L2) unbalanced bilinguals were required to determine whether words belonged to their L1 (i.e., an L1 lexical decision task composed of interlingual homographs, cognates, matched control words from both Chinese and Japanese, and pseudowords). Compared with Chinese-control words, the greater activations in the left IFG and SMA were observed during the processing of interlingual homographs. Although no cognate facilitation effect was observed in terms of reaction time, an interference effect was observed resulting from higher SMA activity during the recognition of cognates. The higher SMA activity suggests that unbalanced bilinguals may face response conflicts when recognizing cognates, even if the task requires them to decide on their dominant language (L1) under the mixed language context. Taken together with van Heuven et al. (2008) mentioned earlier, the IFG may be associated with the stimulus conflict caused by the cross-linguistic ambiguity from different semantic and phonological representations in two languages (e.g., interlingual homographs), and the SMA/dACC is likely associated with the response conflict at the response level. These previous neuroimaging studies indicate that both stimulus list composition and task requirements affect the interference resulted from the non-target language.

It remains unclear whether the discrepancy between bilinguals’ L1 and L2 proficiency levels can modulate the degree of the response conflicts during the recognition of cognates. The lexicons of bilinguals’ two languages are integrated (Dijkstra and van Heuven, 1998, 2002). When unbalanced bilingual individuals access their L2 words, the dominance of the L1 accelerates the recognition of cognates in the L2 through the connection between the shared lexical representation. For this reason, the cognate facilitation effect is greater in the L2 than in the L1 (Brenders et al., 2011), particularly among unbalanced bilinguals (Pérez et al., 2010; Bultena et al., 2013; cf. Rosselli et al., 2014; Robinson Anthony and Blumendeld, 2019 for how the results were influenced by different language dominance and proficiency indexes). Although the studies reported that the recognition of cognates could be modulated by the stimulus list composition, participants in those studies were balanced bilinguals or had a high L2 proficiency - for instance, Dutch native speakers who were proficient in English (e.g., Poort and Rodd, 2017; Peeters et al., 2019; Vanlangendonck et al., 2020). Once the participants’ L2 proficiency becomes heterogeneous or intermediate, it is still unclear whether a mixed-language stimulus list composition leads to response conflicts during cognate recognition. This issue has not been explicitly addressed in previous studies, and the present study aims to fill this gap.

This study investigates the following two research questions. First, we attempted to examine which brain system is associated with resolving response conflicts from cross-linguistic word types in a mixed-language context. Second, we were interested in examining how the level of L2 proficiency mediates response conflict resolution. To address these research questions, we asked Chinese–Japanese late bilinguals with different L2 proficiency levels to perform an L2 lexical decision task composed of cognates, interlingual homographs, and matched control words in Japanese and Chinese.

Following Hsieh et al. (2017), we focused on cross-linguistic words (cognates and interlingual homographs) between logographic writing systems: Chinese characters and Japanese kanji. The phonological systems of these two languages are different from each other. Kuo (2015) indicated that Chinese tones and Japanese accents are different from each other. Chinese tones influence representations from monosyllables, such as 師 /shī/ “teacher,” 石 /shiì/ “stone,” 史 /shǐ/ “history,” 市 /shì/ “city,” but what Japanese accent can influence is at least disyllables, such as 桃 /mómo/ “peach” and 腿 /momó/ “thigh.” In addition to consonants (C) and vowels (V), glides (G) [i], [ɥ], and [w], are widely used in Chinese. Both (C)V (e.g., 說 /tǔ/ “soil”) and (C) GV (e.g., 窩 /wō/ “nest”) are acceptable phonological structure in Chinese, but (C)V is the only acceptable phonological structure in Japanese (e.g., 腿 /momó/ “thigh”). These phonological discrepancies are not the same as previous studies on cognates in Indo-European languages. The phonological overlap is also observed from cognates when the two languages belong to the same language family (e.g., Dijkstra et al., 2010; Comesaña et al., 2012). Even though the phonological systems are different between Chinese and Japanese, the facilitation effect occurs in recognizing cognates (Xiong et al., 2020). This is because the Japanese language employs Chinese characters to represent semantic meanings. With these cross-linguistic word types in a mixed-language stimulus list composition, we can discuss whether response conflicts are resolved outside the word identification system. We formed two different hypotheses for cognates and interlingual homographs.

(1) Cognates: because of the orthographic and semantic overlap in Chinese and Japanese (e.g., 銀行 means “bank” in both Chinese and Japanese) in cognates, stimuli conflicts would not occur in the word identification system. However, providing both L1 and L2 words in the stimuli composition list may enhance participants’ awareness of both language memberships. This awareness would cause interference from the non-target language membership (i.e., L1) to respond to the target language in the L2 lexical decision task.
(2) Interlingual homographs: the orthographic forms are shared by Chinese and Japanese in interlingual homographs. However, their meanings are not (e.g., 汽車 means “car” in Chinese, but it means “train” in Japanese). The different meanings between Chinese and Japanese would lead to stimulus conflict in the word identification system, as observed by Hsieh et al. (2017). Furthermore, the task requirement (i.e., L2 lexical decision task) may increase response conflict in the task/decision system because the non-target language (L1)’s representations of IH words enhance a tendency for the response to ‘no’ response for L2 lexical decision [see van Heuven et al., 2008 for the increased response conflict for IH words in the English (L2) lexical decision task].

We argue that resolving response conflicts is essential when bilinguals recognize cognates in a mixed-language context. Bilinguals’ two languages are simultaneously accessed in the word identification system, and the stimulus list composition would enhance their awareness of language memberships. Because of the explicit response required to differentiate between two languages, the shared orthography and meaning of cognates should establish connections with both “YES” (i.e., accept cognates as L2 words) and “NO” responses (i.e., reject cognates because they are L1 words). To match the task requirement, participants have to inhibit the link between the L1 membership and the “NO” response during cognate recognition. Following the BIA+ model and Multilink, we hypothesized that the mixed-language stimulus list composition would enhance the response conflicts in the task/decision system. The advantage of cognates would be negated in the mixed-language context due to co-activation of language memberships when bilinguals encounter cross-linguistic overlap orthography (Peeters et al., 2019).

To clarify the extent to which word identification and the task decision system are involved in cognate recognition in the brain, we focused on the following brain areas as ROIs: the left IFG, left insula, and SMA. In addition to lexical decision tasks, the activation of the SMA has been reported by studies related to decision making, particularly when participants faced challenging conditions (Duncan, 2010; Camilleri et al., 2018). Thus, the SMA may be involved in resolving the response conflict in the task/decision system during bilingual word recognition. Recent meta-analytical studies also suggest that late bilinguals rely on the left insula and left IFG to process lexico-semantic information in their L2 (Liu and Cao, 2016; Sulpizio et al., 2020). In particular, left IFG activation may be related to the semantic process (see Friederici and Gierhan, 2013 for a review). Thus, both the left IFG and left insula are likely to be associated with the stimulus conflict from lexical representations in the word identification system. In our experiment, the activation of the SMA may be shared by cognates and interlingual homographs because resolving response conflicts is essential. However, the activation of the left IFG and left insula may be associated with processing interlingual homographs, which elicit stimuli conflicts in the word identification system.

Based on the neuroimaging evidence of cognate processing, we expected that the brain areas associated with the word identification system and task/decision system would react differently. It is essential to resolve response conflicts in the task/decision system during cognate recognition. Considering the bilinguals’ L2 proficiency levels, we expected that the level of L2 proficiency could lead to significant differences between the task/decision system and word identification system during L2 word processing. By examining the issues mentioned above, the current study would deepen our understanding of neural correlates of bilingual word recognition.

## Materials and Methods

### Participants

The bilingual participants were similar to those used in Hsieh et al. (2017) and included 22 right-handed Taiwanese individuals who were native speakers of Chinese and had learned Japanese as an L2 [13 males; mean (±*SD*) age: 24.64 ± 3.47; mean age of acquisition (±*SD*) in Japanese: 20.72 ± 3.56]. All bilingual individuals were required to take the Japanese Language Proficiency Test N2 (JLPT N2; the Japan Foundation and Japan Educational Exchanges and Services) before the functional magnetic resonance imaging (fMRI) experiment. These 22 Chinese–Japanese late bilinguals participated in both experiment sessions of Hsieh et al. (2017) and the current study. The experiments were divided into different sessions, and the stimuli were counterbalanced across participants in the experiments. The difference between Hsieh et al. (2017) and the current study was that we further selected participants for data analysis, depending on (1) the scores from the JLPT N2 and (2) the accuracy rates of each word type in the L2 lexical decision task during the fMRI experiment (i.e., cognates, interlingual homographs, matched Japanese and Chinese control words, and pseudowords). To ensure that bilinguals had sufficient Japanese proficiency in recognizing Japanese lexical items, we selected participants who had achieved more than 50% accuracy rates in the JLPT N2. Besides, the participants had to achieve more than 60% total accuracy in the lexical decision task. The accuracy rates of each word type had to be at least 50%. This study was conducted with the approval of the institutional review board of the Graduate School of Medicine, Tohoku University, Sendai, Japan. Written informed consent was obtained from each participant before scanning.

### Stimuli

Stimulus list composition in the experiment was identical to that used in Hsieh et al. (2017). It comprised a mixed-language stimulus list including five types of two-character words—cognates (COs), interlingual homographs (IHs), control words from either Chinese (CCs) or Japanese (JCs), and pseudowords (PWs) that did not exist in either Chinese (L1) or Japanese (L2). A considerable number of earlier studies included different levels of cross-linguistic orthographic overlapped cognates in the experimental list. This methodology may result in limitations in the earlier studies. According to BIA+ and Multilink, different levels of cross-linguistic orthographic similarity lead to different patterns of word recognition because they rely on different representations during bilingual word recognition (Dijkstra et al., 2010; Comesaña et al., 2015). To avoid the involvement of different levels of orthographic similarity between languages, we selected only words that have identical orthographic forms in both Traditional Chinese and Japanese. For instance, although both 大學 (Chinese) and 大学(Japanese) are translated as “university” in the respective languages, such words were not selected as stimuli owing to the different orthographic forms (i.e., 学 and 學). Unlike the intrinsic connection between orthography and phonology in the alphabetic writing system, the orthographic overlap does not lead to the phonological overlap between Chinese and Japanese (Xiong et al., 2020). The widely accepted view is that logographic writing systems are recognized predominantly through the mapping between orthographic and lexical-semantic representations rather than phonological information (see Zhou et al., 2009 for a review). Although some studies mentioned that phonological information is activated earlier than semantics in logographic writing systems (e.g., Perfetti et al., 1995), this view was challenged. For instance, Han and Bi (2009) reported that a patient suffering from phonological deficits still understood the meaning of visually presented Chinese characters. This lesion study suggests that access to phonological information is not the priority for recognizing logographic writing systems. Instead, the mapping between orthographic and lexical-semantic representations is.

In this present study, COs share both the orthographic form and meaning in Chinese and Japanese (e.g., 教室 means *classroom* in both Chinese and Japanese). IHs carry identical orthographic forms but different meanings in Chinese and Japanese (e.g., 麻雀 means *sparrow* in Chinese but *mahjong* in Japanese). Using corpora (ChineseTaiwanWac and JpWac) on Sketch Engine^1^, we controlled the objective frequency across word types in both Chinese (mean: CO = 57.99; IH = 43.74; CC = 36.59 per million, *F*[2,217] = 1.129, *p* = 0.325) and Japanese (mean: CO = 39.92; IH = 30.62; JC = 31.33 per million, *F*[2,217] = 0.500, *p* = 0.608). To ensure the participants’ familiarity with the stimuli, we further invited Chinese and Japanese native speakers who did not participate in this experiment to complete a seven-point scale rating test, and no significant difference was observed among the real words in Chinese [mean: CO = 6.55; IH = 6.47; CC = 6.66, *F*(2,63) = 0.639, *p* = 0.531] and Japanese [mean: CO = 6.30; IH = 6.32; JC = 6.49, *F*(2,63) = 0.669, *p* = 0.516]. We further controlled the number of strokes across conditions to prevent interference from visual complexities of logograms [mean: CO = 18.92; IH = 18.46; CC = 18.78; JC = 18.15. Chinese characters: *F*(2,217) = 0.115, *p* = 0.892; Japanese kanji characters: *F*(2,217) = 0.384, *p* = 0.681].

Logograms in Chinese and Japanese kanji characters can be combined into different characters, such as phono-semantic compound characters (e.g., 油 “oil” is pronounced as/yóu/because of the character 由 /yóu/ “from”). We followed the orthographic rules of these two writing systems to design pseudowords (PW). For instance, both Chinese and Japanese native speakers could recognize the following logograms: 日 “sun,” 火 “fire,” 口 “mouth,” and 平 “flat.” However, the logograms 炚 and 呯 and the combination 呯炚 carry no meaning in either Chinese or Japanese. Finally, 340 stimuli were prepared for the experiments (CO: 120 words, IH: 60 words, CC: 60 words, JC: 60 words, and PW: 60 words). Half of the stimuli were used in the present study (170 stimuli; CO: 60 words, IH: 30 words, CC: 30 words, JC: 30 words, and PW: 30 words), and the other half were used in Hsieh et al. (2017). The dominant proportion of cognates in this study reflected the situation in real life. According to Xiong et al. (2020), approximately 1,163 of 2,058 two-character words are cognates between Chinese and Japanese. The two sets were counterbalanced across participants in the two experiments.

### Task

**FIGURE 1:**
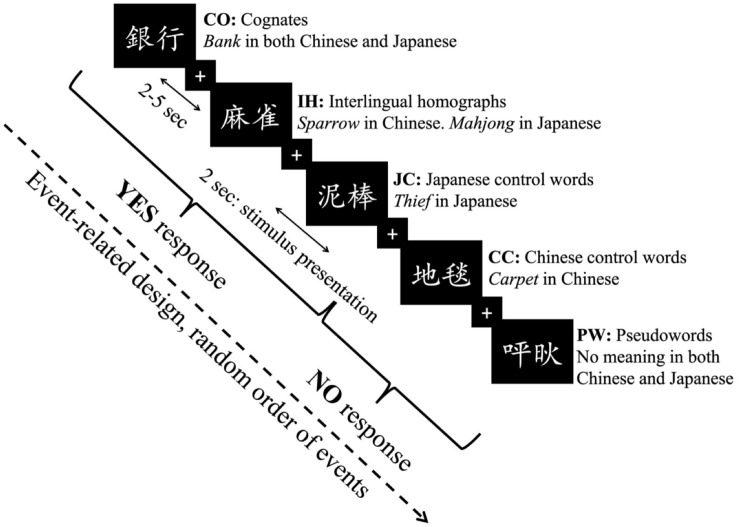
Experimental design. Event-related design for the fMRI experiment.

In the fMRI experiment, all participants were required to complete an L2 lexical decision task. They were asked to determine whether the stimuli belonged to their L2 (Japanese) from among the real word types (COs, IHs, CCs, and JCs) and PWs. Both written and oral instructions in the experiment were provided to the participants in their L1 (i.e., Chinese), but the participants were not informed about the differences among word types in the lexical decision task. If the stimuli belonged to their L2, the participants were instructed to press the “YES” button on the reaction box with the index finger of their right hand to accept them. By contrast, they pressed the “NO” button with their left hand to reject them. Because COs, JCs, and IHs are real words in Japanese, bilinguals were expected to accept them with the “YES” button. On the other hand, they were expected to use the “NO” button to reject CCs and PWs explicitly because these are not Japanese words (Figure 1). Through the mixed-language stimulus list composition, the explicit “NO” response to non-target words not only enhanced the competition between L1 and L2 cognate memberships but also led to the inhibition of L1 membership to achieve task success (Poort and Rodd, 2017; Peeters et al., 2019; Vanlangendonck et al., 2020).

We applied an event-related design to this experiment. Each trial began with presenting a white fixation point (+) on a black background for 2–5 s randomly, and each stimulus lasted for 2 s. The participants were required to make decisions for each stimulus within 2 s. The experiment lasted 900 s in total. Before the fMRI experiment, participants were instructed to minimize their head movements during scanning and practiced ten trials inside the scanner to familiarize themselves with the experimental procedure. E-Prime 2.0 (Psychology Software Tools, Inc.) was applied to present the stimuli and record the buttons pressed by the participants and their reaction times during the experiments.

### Data Analysis of Lexical Decision Task

Sixteen of the 22 participants were included in the final analysis based on their head movements (less than 3 mm), L2 proficiency as assessed by the JLPT test score (over 50%), and accuracy rate of the lexical decision task across all conditions (over 60% in the lexical decision task, with at least 50% in each word type). Four participants were excluded because they had accuracy rates that were lower than 50% in terms of L2 proficiency. Two participants were excluded because their head movements were greater than 3 mm. The selected participants’ accuracy rate in the lexical decision task is 81.16 ± 5.98%.

When analyzing the participants’ reaction times, we focused on the recognition of YES conditions (i.e., COs, JCs, and IHs). In contrast, NO conditions (i.e., CCs and PWs) functioned as fillers to mix stimuli in the L2 lexical decision task. Incorrect responses were excluded because the experimental results might have been influenced. To investigate whether these L2 words (COs, IHs, and JCs) lead to different effects in bilingual word recognition, we used the library *rstatix* (Kassambara, 2020) implemented in R (R Development Core Team^2^) to perform a repeated-measures one-way analysis of variance (ANOVA) on the reaction times (RT) and accuracy rates concerning L2 words. The degrees of freedom were Greenhouse–Geisser-corrected if sphericity was violated. We took L2 proficiency levels into consideration, as they may influence the recognition of L2 words. Through the library *car* (Weisberg, 2019) implemented in R, we used the bilinguals’ JLPT scores as covariates to perform a one-way analysis of covariance (ANCOVA) on the reaction times and accuracy rates for L2 words, separately.

### Neuroimaging Data Acquisition and Preprocessing

Scanning was performed using a 3.0-T Philips Achieva system (Eindhoven, Netherlands). Functional images were acquired with the following parameters: echo time = 30 ms, flip angle = 90°, slice thickness = 3.75 mm, field of view = 240 mm, and matrix = 64 × 64. Every 2 s, 34 axial slices covering the entire brain were obtained, and 437 volumes were acquired for each participant after stabilization of the T1 saturation effect. T1-weighted anatomical images were obtained from each participant to serve as a reference for anatomical correlates (thickness = 1 mm, field of view = 256 mm, matrix = 192 × 224, repetition time = 1,900 ms, echo time = 3.93 ms). The preprocessing procedure was completed using Statistical Parametric Mapping software (SPM12, Wellcome Department of Imaging Neuroscience, London, United Kingdom) in MATLAB (Mathworks, Natick, MA, United States). First, functional volumes were spatially realigned to the first echo-planar imaging (EPI) volume. Then the anatomical T1 image was co-registered to the mean EPI image, which had been generated during the realignment step. Second, the deformation field parameters generated during the normalization of the anatomical T1 image were applied to spatially normalize all EPI scans to Montreal Neurobiological Institute (MNI) space. Third, the original image resolutions of different images (3 mm × 3 mm × 3 mm for EPI images) were smoothed spatially with an 8-mm full-width-at-half-maximum isotropic Gaussian kernel.

### fMRI Analysis

SPM12 was used to conduct a conventional two-level analysis. In the first-level analysis, considering both lexical access and decision making in this experiment, we defined the onset time as when stimuli were presented, and the duration was set as the period of stimuli presentation (2 s). The participants’ functional imaging data were analyzed using a general linear model to measure hemodynamic responses. Only correct responses were accepted in the data analysis, as incorrect responses might influence the experimental results. Five regressors from each word type (CO, IH, JC, CC, and PW) were created for the hemodynamic response model, six-movement parameters (three transitions and three rotations) were included as regressors of no interest, and a high-pass filter with a cut-off period of 128 s was used to eliminate a low-frequency trend. In the second-level analysis, to determine which brain areas were involved in resolving conflicts in both the word identification and task/decision systems, a repeated-measures ANOVA was performed with L2 words (the YES response conditions: COs, IHs, and JCs).

First, the [IH > CO] and [IH > JC] contrasts were tested to examine brain areas involved in both stimulus conflict and response conflict. Following the BIA+ model’s assumptions (Dijkstra and van Heuven, 2002), because IHs have different meanings in Chinese and Japanese, we assumed that the bilinguals should resolve the stimulus conflict in the word identification system. The response conflict in the task/decision system would be further enhanced because the two different meanings led to competition between languages. Although both IHs and COs existed in Chinese and Japanese, only IHs required the resolution of different meanings between languages. Therefore, the [IH > CO] contrast may further elucidate the cognitive demands of stimulus conflict resolution (i.e., semantic representations) between two cross-linguistic word types. Furthermore, the task decision system may be affected by contextual information (e.g., stimulus list composition) and eliminate the link between responses and representations of the non-target language during IH recognition (see Dijkstra and van Heuven, 2018 for a review).

Second, we tested the [CO > JC] contrast to identify brain areas associated with the response conflict elicited by the awareness of language memberships. In the mixed-language stimulus list composition, the L1 control words might have enhanced the participants’ awareness of language membership. When the participants processed COs, the shared orthographic forms and meanings between L1 and L2 might have linked the COs to the “NO” response (i.e., view COs as L1 words), further leading to the response conflict in the task/decision system because it was assumed that participants would eliminate the link between COs and the “NO” response to fulfill the task requirement.

Third, we conducted ROI analyses to elucidate the brain areas involved in word identification and task/decision systems. Because the word identification system is associated with lexical access, we used ROIs comprising 15-mm spheres centered at the left triangular part of the IFG [−46, 32, −14] and the left insula [−32, 26, −6], as defined by a recent meta-analysis (Sulpizio et al., 2020). The left IFG was reported to be related to semantic processing (see Friederici and Gierhan, 2013 for a review), and activation of the left IFG and left insula was related to unbalanced bilinguals’ L2 processing (Liu and Cao, 2016; Cargnelutti et al., 2019). For the task/decision system, because the SMA is reportedly related to decision making that requires high cognitive control (Duncan, 2010; Camilleri et al., 2018), we focused on the ROI comprising a 15-mm sphere centered at the SMA [−6, 20, 49], as defined by Hsieh et al. (2017), which is directly related to this study. For the ROI analyses, we extracted the parameter estimates for the brain mentioned above areas under each word condition (IH, CO, and JC) for each participant using the Marsbar toolbox (Brett et al., 2002). Repeated-measures ANOVA was performed according to word type (i.e., CO, IH, and JC) for the left IFG, left insula, and SMA. Log transformation was applied if the dataset did not meet the normality assumption based on the Shapiro-Wilk test (i.e., the ROI data set from SMA in this study). The degrees of freedom were Greenhouse–Geisser-corrected if sphericity was violated.

Finally, as with the behavioral data, we also used the bilinguals’ JLPT scores as covariates to perform one-way ANCOVA on brain activation levels in the left IFG, left insula, and SMA to determine whether L2 proficiency influenced the word identification and task/decision systems.

## Results

### Behavior Results

#### Lexical Decision Task

To investigate the existence of the cognate facilitation effect, we analyzed the mean RTs and accuracy rates related to the L2 words (COs, IHs, and JCs). The mean RTs and accuracy rates are summarized numerically in Table 1 with the responses for each word type.

**TABLE 1:** Mean reaction times (RT) and accuracy rates associated with each word type.

Required response in the Japanese (L2) task	Types of words	RT ms (*SD*)	Accuracy% (*SD*)
“YES” response	Cognates (CO)	1220 (172)	75.1% (11)
	Japanese control words (JC)	1034 (177)	94.4% (10)
	Interlingual homographs (IH)	1273 (190)	59.4% (8)
“NO” response	Chinese control words (CC)	1322 (192)	79.1% (12)
	Pseudowords (PW)	965 (212)	97.9% (5)

The one-way ANOVA revealed a significant difference in RT [*F*(2,30) = 51.23, *p* < 0.001]. *Post hoc* pairwise comparisons showed significantly longer reaction times for COs than for JCs (*t* = 9.01, *p* < 0.001), and this pattern was also observed between IHs and JCs (*t* = 7.94, *p* < 0.001). However, no difference was observed between COs and IHs (*t* = −2.36, *p* = 0.97).

Regarding the accuracy rate, a significant difference was found among the word types [*F*(2,30) = 74.02, *p* < 0.001]. *Post hoc* pairwise comparisons showed a significantly lower accuracy rate for IHs than for COs (*t* = 6.05, *p* < 0.001) and JCs (*t* = −11.08, *p* < 0.001). Moreover, the accuracy rate for COs was significantly lower than that for JCs (*t* = −6.28, *p* < 0.001).

### Role of L2 Proficiency in Word Recognition

**FIGURE 2:**
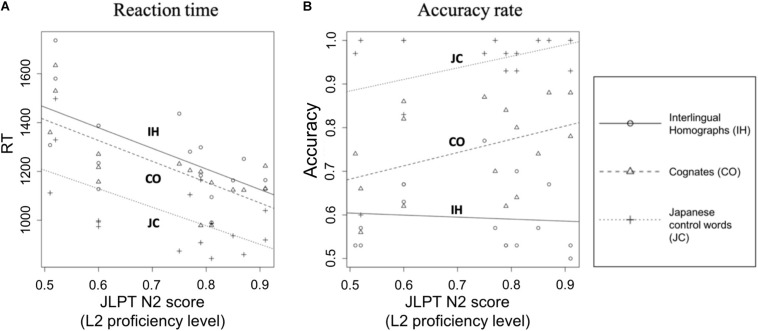
Relationship of proficiency level with participants’ mean RT and mean accuracy rate. In **(A)**, the *x*-axis indicates the bilinguals’ proficiency level. The *y*-axis indicates the mean RT to each L2 word type (CO, cognate; JC, Japanese control word; IH, interlingual homograph). The proficiency level was consistently related to the response to Japanese words. Higher proficiency levels were associated with faster RTs. In **(B)**, the *y*-axis indicates the bilinguals’ mean accuracy rate for each L2 word type. The RT tendency in **(A)** was not observed in the accuracy rate data. Higher proficiency levels were associated with a relatively low accuracy rate for IHs in comparison with COs and JCs.

We conducted additional analyses to investigate whether the recognition of L2 words (COs, IHs, and JCs) was determined by the level of L2 proficiency. We performed a one-way ANCOVA on the bilinguals’ RTs and accuracy rates with each bilingual’s L2 proficiency score (JLPT N2) as a covariate. For RT, there was no interaction between the types of words (COs, IHs, and JCs) and L2 proficiency [*F*(2,42) = 0.04, *p* = 0.96]. The main effect of types of words was significant [L2 word type: *F*(2,44) = 13.3, *p* < 0.001]. The main effect of L2 proficiency was significant [*F*(1,44) = 32.7, *p* < 0.001]. Significant correlations were observed between bilinguals’ RT of each word type and individual’s L2 proficiency levels [COs: *t*(13) = −3.13, *r* = −0.66, *p* < 0.01; IHs: *t*(13) = −2.37, *r* = −0.55, *p* < 0.05; JCs: *t*(13) = −2.16, *r* = −0.51, *p* < 0.05]; that is, bilinguals with higher Japanese proficiency could recognize COs, IHs, and JCs faster (Figure 2A). Regarding accuracy rates (Figure 2B), we did not observe an interaction between the types of words and L2 proficiency [*F*(2,42) = 1.28, *p* = 0.29]. The main effect of types of words was significant [*F*(2,44) = 54.9, *p* < 0.001], but the main effect of L2 proficiency was not significantly related to their accuracy rates [*F*(1,44) = 3.13, *p* = 0.08]. No correlation was observed between each word type and L2 proficiency levels, except for COs, which presented a marginally significant correlation with L2 proficiency levels (*S* = 403.65, ρ = 0.41, *p* = 0.059).

### Brain Imaging Results

**FIGURE 3:**
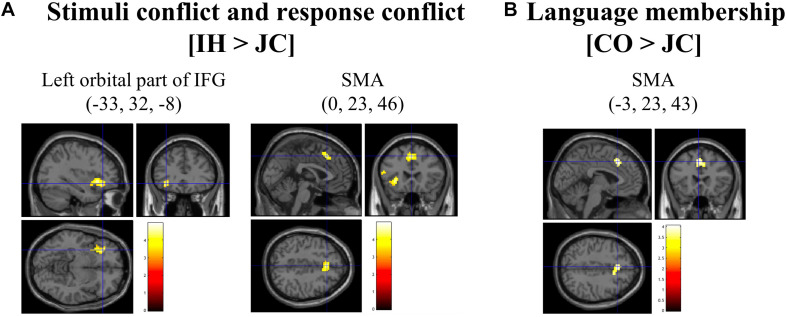
Brain areas observed by comparisons between types of words. In **(A)**, the contrast of [interlingual homograph (IH) > Japanese control word (JC)] revealed significant activation of the left orbital part of the IFG and the supplementary motor area. In **(B)**, the contrast of [cognate (CO) > Japanese control word (JC)] revealed activation in the supplementary motor area. The threshold was set at *p* < 0.05 after cluster-level correction (initial voxel-level height threshold, *p* < 0.001).

Brain activations for cross-linguistic effects are recorded in Table 2. First, the contrast of [IH > CO], which was associated with semantic conflict, did not reveal any significant activation under the liberal threshold (uncorrected for voxel-level, *p* < 0.001). Second, the contrast of [IH > JC] showed significant activation in the left orbital part of the IFG and the SMA (Figure 3A). Third, the greater activation in the SMA was observed in the contrast of [CO > JC] (Figure 3B).

**TABLE 2:** Brain activation patterns associated with cross-linguistic effects.

Structure	*x, y, z*	*T*	Cluster size
Stimulus conflict and Response conflict Interlingual homographs > Cognates [IH > CO] *No significant brain activation*			
Stimulus conflict and Response conflict Interlingual homographs > Japanese control words [IH > JC]			
Left orbital part of the IFG	−33, 32, −8	4.95	194
	−33, 20, −5	4.59	
	−45, 20, −2	3.98	
Supplementary motor area	0, 23, 46	4.44	121
Language membership Cognates > Japanese control words [CO > JC]			
Supplementary motor area	−3, 23, 43	4.04	99
	6, 26, 37	3.68	
	9, 14, 46	3.62	

**The coordinates (x, y, z) of the activation peak in MNI space, peak T-value, and size of the activated cluster for Chinese–Japanese bilinguals (n = 16) are presented for each ROI. The threshold was set after cluster-level correction at p < 0.05 (initial voxel-level height threshold, p < 0.001)*.*

**FIGURE 4:**
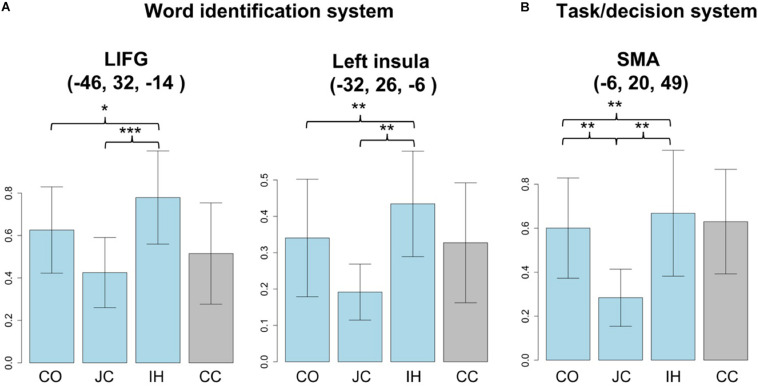
Activation patterns associated with the response to each word type. The *x*-axis indicates the word types (CO, cognate; JC, Japanese control word; IH, interlingual homograph; CC, Chinese control word). The *y*-axis indicates the mean activation level during the response to each word type. The response to pseudowords (PWs) was treated as the baseline. **(A)** A significant difference was observed between the responses to IHs and JCs in the word identification system, LIFG (left inferior frontal gyrus) and left insular. **(B)** In the task/decision system, SMA (supplementary motor area), a significant difference was observed not only between the responses to IHs and JCs but also between those to COs and JCs (*p < 0.05, **p < 0.01, ***p < 0.001).

To clarify different activation patterns in the word identification and task/decision systems, we used ROIs comprising 15-mm spheres centered at the left triangular part of the IFG [−46, 32, −14], the left insula [−32, 26, −6] (word identification system), and the SMA [−6, 20, 49] (task/decision system), as defined by recent studies (Hsieh et al., 2017; Sulpizio et al., 2020). Activation patterns were further extracted to compare cognitive processing for each word type (Figure 4). In the ROIs for the word identification system, significant differences were observed in the response to the types of words [left triangular part of IFG: *F*(2,30) = 5.119, *p* < 0.05; left insula: *F*(2,30) = 5.47, *p* < 0.01]. The *post hoc* pairwise test further indicated differences between IHs and JCs (left triangular part of the IFG: *t* = 4.27, *p* < 0.001; left insula: *t* = 4.02, *p* < 0.01) but not between COs and JCs in both the left triangular part of the IFG (*t* = 1.59, *p* = 0.396) and the left insula (*t* = 1.75, *p* = 0.101). For the task/decision system, because the datasets about SMA responses to IHs and COs did not match the null hypothesis of the Shapiro–Wilk test (*p* < 0.05), we log-transformed the datasets to meet the normality assumptions of the Shapiro–Wilk test. A significant difference was observed in response to the types of words [*F*(2,30) = 5.806, *p* < 0.01]. Similar to responses in the brain areas for the word identification system, the *post hoc* pairwise test indicated a difference between the responses to IHs and JCs in the SMA (*t* = 3.07, *p* < 0.01). Moreover, a significant difference was observed between the responses to COs and JCs (*t* = 3.15, *p* < 0.01). No significant difference was observed between the responses to IHs and COs from the aforementioned peaks (left triangular part of the IFG: *t* = −1.29, *p* = 0.217; left insula: *t* = −1.26, *p* = 0.226; SMA: *t* = −0.467, *p* = 0.647). When we performed an ROI analysis based on the coordinate of the pre-SMA/ACC (−2, 16, 54) in van Heuven et al. (2008), the results are the same as the present ROI analysis [*F*(2,30) = 4.65, *p* < 0.05. IHs vs. COs: *t* = −0.354, *p* = 1.00; IHs vs. JCs: *t* = 2.81, *p* < 0.05; COs vs. JCs: *t* = 3.43, *p* < 0.05].

### Role of L2 Proficiency in the Bilingual Brain

**FIGURE 5:**
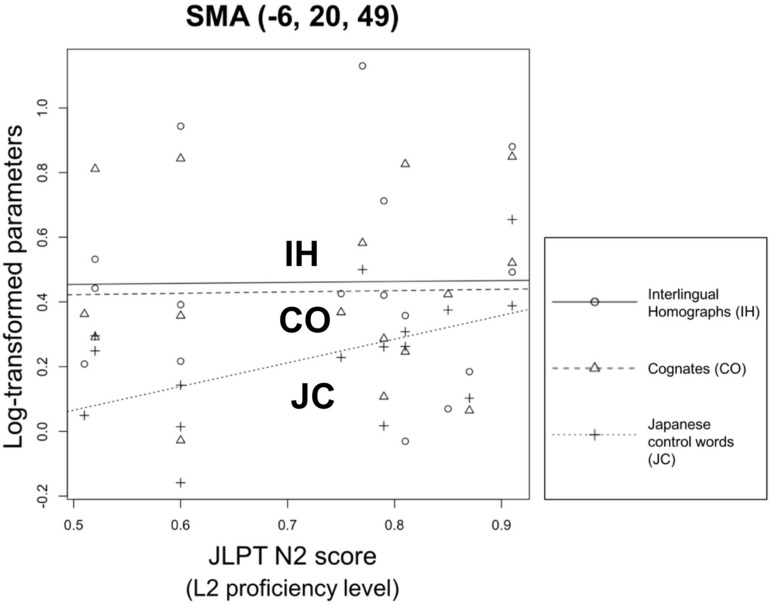
Relationship between participants’ proficiency level and response to types of words in the SMA. The *x*-axis represents the bilinguals’ L2 proficiency level. The *y*-axis indicates the log-transformed response data of the SMA activity level. Even though significant difference could be observed from the types of words (IH, CO, and JC), the effect of L2 proficiency is inconsistent among three types of words [*F*(1,44) = 0.88, *p* = 0.35].

To determine whether L2 proficiency influences brain activation patterns in word identification and task/decision systems during lexical decision tasks, we performed one-way ANCOVA on the bilinguals’ brain activities in the left triangular part of the IFG, left insula, and SMA with their JLPT N2 scores as a covariate. In the aforementioned areas, no interaction was observed between the response to the types of words (COs, IHs, and JCs) and L2 proficiency [left triangular part of the IFG: *F*(2,42) = 0.64, *p* = 0.53; left insula: *F*(2,42) = 0.87, *p* = 0.43; SMA: *F*(2,42) = 0.6515, *p* = 0.5265]. In the areas responsible for the word identification system (i.e., left triangular part of the IFG and left insula), only marginally significant effects were observed in the response to the types of words [left IFG: *F*(2,44) = 3.04, *p* = 0.058; left insula: *F*(2,44) = 3.17, *p* = 0.052]. For the area responsible for the task/decision system (i.e., SMA; Figure 5), significant differences were observed in the log-transformed datasets of responses to the types of words [*F*(2,44) = 3.36, *p* < 0.05]. However, the main effect bilinguals’ L2 proficiency levels was not significant in each brain area of ROI [left IFG: *F*(1,44) = 0.04, *p* = 0.84; left insula: *F*(1,44) = 0.004, *p* = 0.95; SMA: *F*(1,44) = 0.88, *p* = 0.35].

## Discussion

To our knowledge, this is the first study using fMRI to investigate cognitive processing of logographic cognates in a specific language lexical decision task involved a mixed language list. In contrast to previous studies discussing this issue using a single language stimulus list composition, we observed the absence of a cognate facilitation effect. Cognate recognition relies on task decision systems, although the cognates used here have identical orthographic forms and meanings in Chinese and Japanese.

### Absence of a Cognate Facilitation Effect

The Chinese–Japanese bilinguals took significantly longer to recognize IHs than JCs. In comparison with other word types, the accuracy rate of IHs was expected to be lower due to the interference from the non-target language (i.e., L1). Neuroimaging results also indicate the activation of the left orbital part of the IFG and SMA when recognizing IHs. The activations of the left IFG and SMA, reported being language control areas (Abutalebi and Green, 2016), align with the results of resolving stimuli conflicts and response conflicts (van Heuven et al., 2008; Hsieh et al., 2017; Peeters et al., 2019). According to van Heuven et al. (2008), stimuli conflicts are observed from IHs because (1) they belong to two languages, and (2) they are semantically ambiguous. Response conflicts are also enhanced when bilinguals need to respond appropriately (i.e., determine whether the stimuli belong to a certain language). When recognizing IHs, it is necessary to resolve the conflicts arising from different meanings in Chinese and Japanese in the word identification system (van Heuven et al., 2008; Hsieh et al., 2017). Moreover, the activation of the SMA has been linked to response conflicts requiring high cognitive control (Duncan, 2010; Camilleri et al., 2018; Chien et al., 2020). In sum, the Chinese–Japanese bilinguals might have encountered stimuli conflicts and response conflicts when recognizing IHs during the lexical decision task.

In contrast to previous studies using a single language stimulus list (e.g., Dijkstra et al., 2010; Xiong et al., 2020), the cognate facilitation effect was absent in this study. Although cognates share both orthographic form and meaning between Chinese and Japanese, the bilinguals took longer time to recognize cognates than Japanese control words, and no significant difference was observed in the RTs for IHs and COs. In this study, the accuracy rates for COs did not match those of previous studies about cognate facilitation effects, as they were associated with higher error rates than were JCs. Because of the shared orthography and semantics in cognates, stimuli conflicts cannot be observed in the word identification system. The cognitive demand of processing cognates was due to response conflicts, which is supported by the activation of the SMA. The results mentioned above suggest the essence of resolving response conflicts when recognizing cognates during the L2 lexical decision task, especially in a language-specific lexical decision test involved a mixed language stimulus list.

In this study, we used logographic writing systems (Chinese and Japanese kanji characters) to create a mixed-language stimulus list composition including COs, IHs, matched control words specific to either Chinese or Japanese, and PWs in the L2 lexical decision task. The experiment design is different from that of previous studies, which discuss alphabetic writing systems (e.g., Peeters et al., 2013) and logographic writing systems (e.g., Xiong et al., 2020) with a single language stimulus list including only cognates and matched control words from either the L1 or L2. Although the cognate facilitation effect has been frequently reported in earlier studies with a single language stimulus list, whether this is a relevant facilitative effect is still controversial. Because no word belongs to the language other than the target language, any lexical activation can be considered evidence supporting facilitation in word recognition (Vanlangendonck et al., 2020). The experimental design of the current study can foster a more thorough discussion of the cognitive processes activated when bilinguals recognized cognates. Because the bilinguals had to decide between two explicit responses (YES or NO) during the L2 lexical decision task that included a mixed-language stimulus list, a connection between the “NO” response and the L1 orthographic form was likely established. In particular, the participants were unbalanced bilinguals, and the relatively higher subjective frequency of the L1 compared to the L2 likely activated the L1 language membership first when facing COs, further leading to a possible rejection of COs. However, COs should be accepted because they belong to both Chinese and Japanese. Fulfilling the task requirement requires subsequent response inhibition of non-target language membership (Peeters et al., 2019). Both behavioral and neuroimaging results indicate that the recognition of COs in the mixed-language context elicited a cognitive process of resolving response conflicts compared to the response to JCs. First, similar to IHs, COs took more time for recognition compared to JCs. Second, the activation of the SMA, which is the brain area associated with resolving response conflicts, strengthens this viewpoint. The results of this study are consistent with those of previous studies that applied alphabetic writing systems to L2 lexical decision tasks composed of mixed-language stimulus lists (e.g., Poort and Rodd, 2017; Peeters et al., 2019; Vanlangendonck et al., 2020).

### Resolving Response Conflicts Is Outside the Word Identification System

In addition to the absence of a cognate facilitation effect, the ROI analysis further confirms that the response conflict enhanced by language memberships was resolved in the task/decision system. In the brain areas associated with the word identification system (left triangular part of the IFG and left insula), a significant difference was observed between the responses to IHs and JCs, not between those to COs and JCs. Because IHs carry different meanings between Chinese and Japanese, the activation of the left triangular part of the IFG and left insula is essential to resolve the stimulus conflicts enhanced by semantics in the word identification system. This cognitive process is not needed to recognize COs because of the shared meaning in Chinese and Japanese.

In the area for the task/decision system (SMA), in comparison with the response to JCs, significant differences were observed with the responses to both IHs and COs. The activation of the SMA is related to high cognitive control under challenging conditions (Duncan, 2010; Camilleri et al., 2018; Chien et al., 2020). When considering COs, the bilinguals might have faced the same level of response conflicts as with IHs during word recognition. Moreover, in a mixed-language composition list, the language memberships of cross-linguistic words (IHs and COs) are linked to both “YES” and “NO” responses in the task/decision system: the L2 membership (i.e., Japanese) of COs is linked to the “YES” response, and the L1 membership (i.e., Chinese) is linked to the “NO” response in the task/decision system. However, in contrast to IHs, the overlapped orthography and semantics of the COs might have resulted in a response conflict in the bilinguals in the lexical decision task within a mixing language context [e.g., the lexical representations of the non-target language (i.e., Chinese) were more likely to evoke a “NO” response. However, they were not supposed to do so because COs belong to both Chinese and Japanese]. These characteristics do not lead to cognitive facilitation from cognates, but cognates required to resolve response conflicts resulted from the interference of non-target language membership in the task/decision system.

These findings suggest that the task/decision system is responsible for resolving response conflicts elicited by language memberships. According to the assumptions of BIA+ and Multilink, the shared orthography of cognates leads to maximal coactivations of semantic representations and creates a link between the “YES” response and the semantic representations. These advantages lead to the cognate facilitation effects in the single language stimulus list; however, it becomes a disadvantage in the mixed-language list. Once bilinguals’ awareness of language memberships is enhanced upon exposure to a mixed-language stimulus list, response inhibition is essential because both language memberships are considered at the response level, even though the meaning is shared by different languages. Because this study required bilinguals to respond “YES” to the L2 words and “NO” to anything else, the bilinguals should have constructed a task schema to determine whether the stimuli belong to the target language. The lexical decision process receives conflicting information as the connection to both Chinese (L1) and Japanese (L2) memberships (Vanlangendonck et al., 2020). Also, the matched control words from both L1 and L2 enhanced response conflicts when recognizing COs. The orthographic and semantic overlap enhanced the difficulty of making an appropriate response to COs (i.e., accept them as L2 words, or reject them as L1 words), further canceling out the facilitation expected from the orthographic and semantic overlap (Poort and Rodd, 2017). Our neuroimaging results indicate that cognates require resolution of response conflicts outside the word identification system (i.e., the activation of SMA) in bilingual word recognition.

### Engagement of L2 Proficiency

Although the response to COs in a mixed-language stimulus list composition did not reflect a facilitative effect, the COs were still associated with advantages compared to IHs during word recognition. The advantage is related to the L2 proficiency level. The highly proficient bilinguals recognized all L2 words (i.e., IHs, COs, and JCs) faster than less proficient bilinguals in the L2 lexical decision task. The accuracy rates associated with COs were positively correlated with the bilinguals’ proficiency, though the result showed a tendency toward statistical significance (*p* = 0.059). This tendency was not observed for IHs and JCs. The results on accuracy are partially consistent with those of Lemhöfer et al. (2015). Lemhöfer et al. (2015) investigated cognitive processing in German–Dutch bilinguals’ word recognition with near-cognates, Dutch non-cognate homophones, and pseudo-homophones. The inhibitory effect increased with the amount of L2 experience when the bilinguals processed near-cognates. This tendency was not observed for the other word types. The current study used both orthographic-identical cognates and IHs between Chinese and Japanese in a mixed-language stimulus list composition. Although COs have two language memberships, the semantic overlap of the COs might have reduced the processing costs compared to IHs.

When we set the bilinguals’ L2 proficiency level as a covariate, we observed a significant difference among the responses to the types of words (COs, IHs, and JCs) in the SMA, which contributes to the task/decision system, not the word identification system (left triangular part of the IFG and left insula). Although IHs and COs resulted in higher activation levels than JCs in this ROI, the level of L2 proficiency was sensitive to JCs only (see Figure 5). No such tendency was shown in IHs and COs. Because our participants were unbalanced bilinguals, the L1 words’ relatively higher subjective frequency than the L2 words might have activated the L1 representations first when facing IHs and COs. Thus, in the L2 lexical decision task, the essential process for recognizing IHs and COs might have been resolving response conflicts enhanced by the link between responses and L1 representations, which were not directly involved in L2 proficiency.

By comparing the present study’s findings with those of Hsieh et al. (2017), we confirm the role of relative proficiency in L1 and L2 in the task/decision system. We used the same peak of SMA as in Hsieh et al.’s (2017) study, which recruited the same group of participants as those recruited in the present study to extract brain activation patterns upon exposure to each word type. Focusing on L1 word recognition, Hsieh et al. (2017) observed significant different activation in SMA between the responses to COs and IHs. The present study, which focused on L2 word recognition, observed greater SMA activation for both IHs and COs than for JCs (control words), but no differences between IHs and COs. These findings suggest that the conflict originating from language memberships is more apparent in the L2 lexical decision task than in the L1 counterpart. Thus, task demands may modulate the involvement of the SMA (task/decision system) during bilinguals’ word recognition.

## Conclusion

Having explored bilinguals’ L2 word recognition concerning a mixed-language stimulus list composition, we suggest that language membership control operates independently from the word identification system during bilingual word recognition. First, we demonstrated that COs, similar to IHs, elicited an inhibitory effect rather than a facilitative effect upon exposure to the mixed-language stimulus list. Contrast analysis of SMA activation, i.e., [CO > JC], further confirmed that the bilinguals experienced difficulties recognizing COs from a mixed-language stimulus list composition. Because the stimuli comprised words from the bilinguals’ L1 and L2, the awareness of language memberships was likely enhanced, further strengthening the connection between a “NO” response and the L2 membership of the COs. Because the bilinguals needed to remove this connection at the response level to fulfill the task requirement (i.e., accept the L2 membership of COs), the subsequent inhibition of the non-target language (L1) membership was essential. The statements mentioned above were supported by our results on the activation of language control areas and from the ROI analyses. In the left IFG and the left insula, compared with the response to JCs, a significant difference was observed for the response to IHs only. In the SMA, both COs and IHs resulted in higher activation levels than did JCs. These results confirm the difficulties associated with COs in the mixed-language stimulus list, further confirming that response conflicts elicited by language memberships are resolved in the task/decision system rather than the word identification system, which is in line with both BIA+ and Multilink. Second, by setting the proficiency level as a covariate, we observed that only the SMA response was sensitive to the bilinguals’ L2 proficiency. By recruiting the SMA, bilinguals may distinguish languages and inhibit the non-target language membership of COs and IHs. Our findings suggest that controlling language memberships allows efficient bilingual language processing.

**FIGURE 6:**
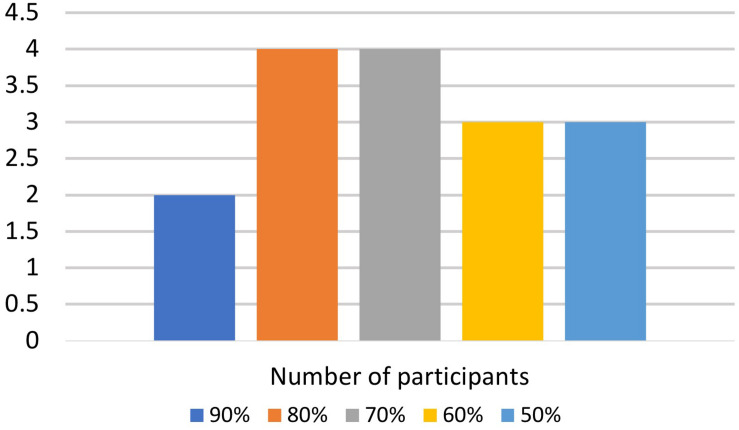
Chinese–Japanese bilinguals’ L2 proficiency level. The limited number of highly proficient bilinguals may be why the L2 proficiency level did not show a consistent effect on the accuracy rate and brain activation patterns.

The current study suggests the importance of the task/decision system when resolving response conflicts enhanced by language memberships during bilingual word recognition within a mixed-language context. When bilinguals are required to control two languages while producing explicit responses, resolving response conflicts becomes essential to respond appropriately to the task requirement. Despite the vital contribution of this study to the field, there are several limitations. First, the number of participants was limited. In order to ensure participants’ quality of L2 response, we selected participants who achieved the following criteria to the data analysis: (1) L2 proficiency level was higher than 50%; and (2) The total accuracy rate in the lexical decision task was above 60%, and the accuracy rate of each word type was higher than 50%. This selecting threshold decreased the number of participants, possibly affecting the replicability (Turner et al., 2018). Second, participants’ L2 proficiency level was limited (see Figure 6). When we set participants’ L2 proficiency level as a covariate in the ANCOVA, it was significantly related to RTs only. The limited number of highly proficient bilinguals (>70%) may be why the L2 proficiency level did not show a consistent effect on the accuracy rate and brain activation patterns. Future studies should consider the large number of late bilinguals achieving advanced L2 proficiency levels. Third, this study focused on participants’ proficiency in the data analysis. However, bilingual experiences such as immersion and code-switching habits might modulate the cognitive process of language control (Luk and Bialystok, 2013). Future studies should focus on this issue and discuss whether the bilingual experience is beneficial for resolving cross-linguistic conflicts. Fourth, although we indicate that the word identification system is separated from the task/decision system, it is still unclear whether the word identification system influences the task/decision system. Future studies will focus on this issue and examine the cause and effect of these two systems. In conclusion, the effect (i.e., null effect or inhibitory effect) resulting from exposure to cognates is not absolute; rather, it depends on exact empirical circumstances, and resolving response conflicts enhanced by language memberships is essential for bilingual word recognition in a mixed-language context.

## Data Availability Statement

The raw data supporting the conclusions of this article will be made available by the authors, without undue reservation, to any qualified researcher.

## Ethics Statement

The studies involving human participants were reviewed and approved by the institutional review board of the Graduate School of Medicine, Tohoku University. The patients/participants provided their written informed consent to participate in this study.

## Author Contributions

M-CH, HJ, MS, and RK conceived and planned the experiments. M-CH prepared stimuli and experimental programming under the supervision of HJ. M-CH and HJ carried out the experiments. M-CH wrote the first draft. HJ, MS, and RK provided feedback and helped to shape the research, analysis, and manuscript. All authors contributed to the article and approved the submitted version.

## Conflict of Interest

The authors declare that the research was conducted in the absence of any commercial or financial relationships that could be construed as a potential conflict of interest.

## Footnotes

1 http://www.sketchengine.co.uk

2 http://www.r-project.org./
